# A luciferase complementation assay system using transferable mouse artificial chromosomes to monitor protein–protein interactions mediated by G protein-coupled receptors

**DOI:** 10.1007/s10616-018-0231-7

**Published:** 2018-07-31

**Authors:** Narumi Uno, Tomohito Fujimoto, Shinya Komoto, Gene Kurosawa, Masaaki Sawa, Teruhiko Suzuki, Yasuhiro Kazuki, Mitsuo Oshimura

**Affiliations:** 1 Department of Biomedical Science, Institute of Regenerative Medicine and Biofunction, Graduate School of Medical Science, Tottori University, 86 Nishi-cho, Yonago, Tottori 683-8503 Japan; 2 Chromosome Engineering Research Center, Tottori University, 86 Nishi-cho, Yonago, Tottori 683-8503 Japan; 3 ProbeX, Inc., 3F BMA, 1-5-5 Minatojima-Minamimachi, Chuo-ku, Kobe, 650-0047 Japan; 4 Department Academic Research Support Promotion Facility, Center for Research Promotion and Support, Fujita Health University, 1-98 Dengakugakubo, Kutsukake-cho, Toyoake, Aichi 470-1192 Japan; 5 Research and Development, Carna Biosciences, Inc., 3F BMA, 1-5-5 Minatojima-Minamimachi, Chuo-ku, Kobe, 650-0047 Japan; 6 Stem Cell Project, Tokyo Metropolitan Institute of Medical Science, 2-1-6 Kamikitazawa, Setagaya-ku, Tokyo, 156-8506 Japan

**Keywords:** Mouse artificial chromosome, SIM system, Split luciferase, GPCR, PTHR2, hEP4, ARRB1

## Abstract

G protein-coupled receptors (GPCRs) are seven-transmembrane domain receptors that interact with the β-arrestin family, particularly β-arrestin 1 (ARRB1). GPCRs interact with 33% of small molecule drugs. Ligand screening is promising for drug discovery concerning GPCR-related diseases. Luciferase complementation assay (LCA) enables detection of protein–protein complementation via bioluminescence following complementation of N- and C-terminal luciferase fragments (NEluc and CEluc) fused to target proteins, but it is necessary to co-express the two genes. Here, we developed LCAs with mouse artificial chromosomes (MACs) that have unique characteristics such as stable maintenance and a substantial insert-carrying capacity. First, an NEluc-ARRB1 was inserted into MAC4 by Cre-loxP recombination in CHO cells, named ARRB1-MAC4. Second, a parathyroid hormone receptor 2 (PTHR2)-CEluc or prostaglandin EP4 receptor (hEP4)-CEluc were inserted into ARRB1-MAC4, named ARRB1-PTHR2-MAC4 and ARRB1-hEP4-MAC4, respectively, via the sequential integration of multiple vectors (SIM) system. Each MAC was transferred into HEK293 cells by microcell-mediated chromosome transfer (MMCT). LCAs using the established HEK293 cell lines resulted in 35,000 photon counts upon somatostatin stimulation for ARRB1-MAC4 with transient transfection of the somatostatin receptor 2 (SSTR2) expression vector, 1800 photon counts upon parathyroid hormone stimulation for ARRB1-PTHR2-MAC4, and 35,000 photon counts upon prostaglandin E2 stimulation for ARRB1-hEP4-MAC4. These MACs were maintained independently from host chromosomes in CHO and HEK293 cells. HEK293 cells containing ARRB1-PTHR2-MAC4 showed a stable reaction for long-term. Thus, the combination of gene loading by the SIM system into a MAC and an LCA targeting GPCRs provides a novel and useful platform to discover drugs for GPCR-related diseases.

## Introduction

Mammalian artificial chromosome vectors, including the human artificial chromosome (HAC) (Kazuki et al. 2011) and mouse artificial chromosome (MAC) (Takiguchi et al. 2012), are suitable systems for stable expression of multiple genes (Oshimura et al. 2014). We previously reported the proof-of-concept of a new system for sequential integration of multiple vectors (SIM) via Cre-loxP recombination and phage integrase/attachment sites, i.e., Bxb1 integrase/attB/attP and PhiC31 integrase/attB/attP, on a HAC (Suzuki et al. 2014). The SIM system is promising for investigation of multiple gene interactions based on circular plasmid vectors.

**Fig. 1:**
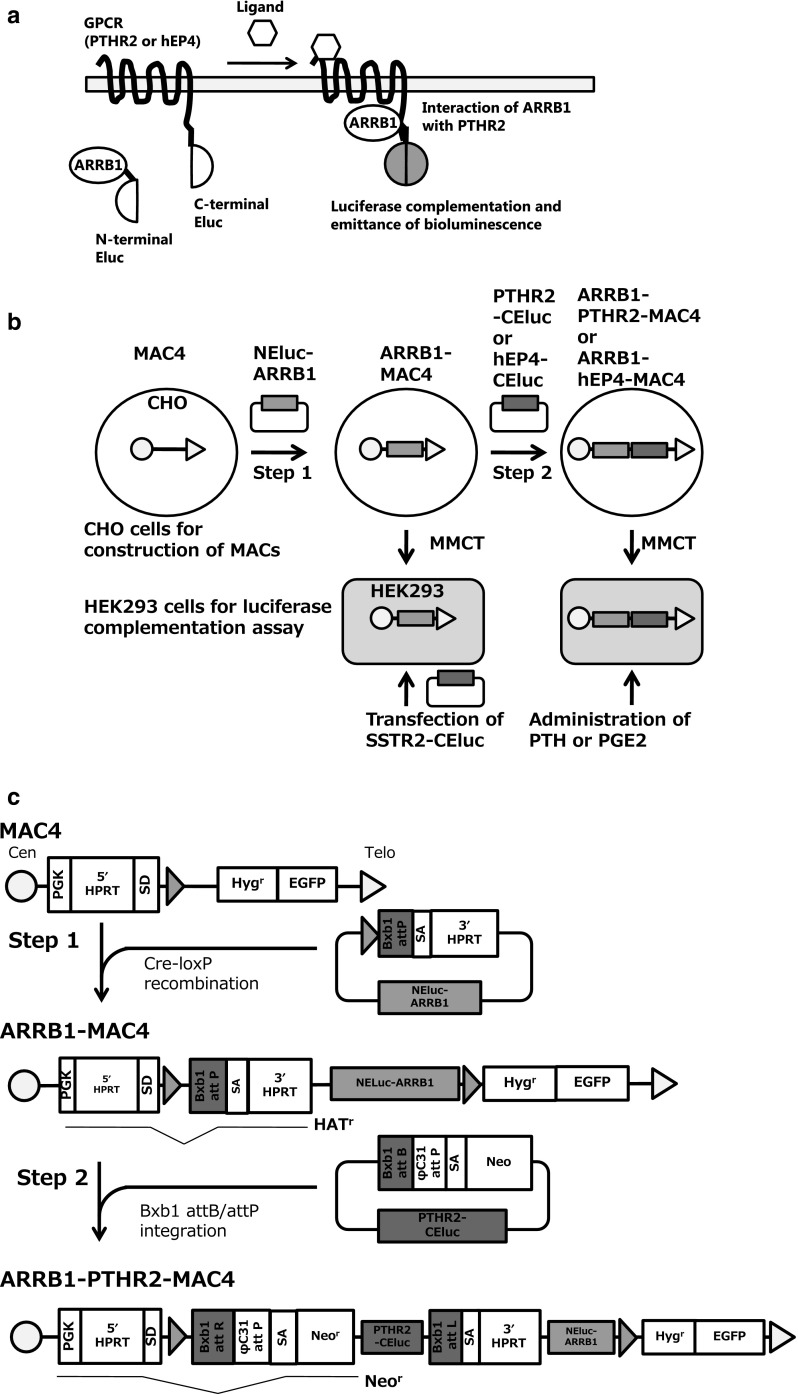
Schematic diagrams of gene loading and evaluation of luciferase activity of GPCRs using split luciferase complementation. **a** A schematic diagram of luciferase complementation of N-Eluc-ARRB1 and PTHR2-CEluc or hEP4-CEluc for evaluation of ligand stimulation activity. **b** Outline of gene loading into MACs and chromosome transfer of the MACs. ARRB1 was loaded at step 1 (ARRB1-MAC4), and then GPCRs-CEluc (PTHR2-CEluc or hEP4-CEluc) were loaded at step 2 into the MAC (ARRB1-PTHR2-MAC4 or ARRB1-hEP4-MAC4). These MACs were transferred into HEK293 cells to evaluate the bioluminescence activity by the LCA via MMCT. The SSTR2-CEluc expression vector was transiently transfected into HEK293 cells to evaluate the bioluminescence activity of ARRB1-MAC4. **c** Construction of ARRB1-GPCR-MAC4 using the SIM system. The SIM system enables insertion of multiple circular vectors sequentially into a MAC. It involves various kinds of site-specific recombination using Cre-loxP, Bxb1 integrase-Bxb1 attB/attP, and ϕC31 integrase-ϕC31 attB/attP. These enzymes and acceptor sites reconstitute selectable markers on the MAC. Therefore, cells acquire resistance to antibiotics when a circular plasmid is inserted into the MAC. First, NEluc-ARRB1 is inserted into MAC4 via recombination with Cre-loxP, and reconstitution of the HPRT gene leads to resistance against HAT medium (Step 1). The inserted plasmid has another specific recombination site, Bxb1 attP, which enables sequential insertion of further circular vectors with Bxb1 integrase. Second, GPCRs-CEluc, which are PTHR2-CElu or hEP4-CEluc, are inserted onto ARRB1-MAC4 via recombination of Bxb1 attB and attP with Bxb1 recombinase. The Neo resistance gene, which has a splicing acceptor site (SA) and traps PGK promoter activity instead of the HPRT gene, shows resistance against G418, and the HPRT gene is disrupted, resulting in cells with sensitivity to HAT medium. Theoretically, the φC31 attP site enables further gene loading (Step 2)

GPCRs comprise a family of seven-transmembrane receptors. They are a unique receptor family targeted by 12% of human proteins and 33% of small molecule drugs that target the major receptor families (Santos et al. 2017). GPCRs sense ligand signals by an extracellular module and then undergo a conformational change that transduces the signal via interactions of the intracellular module and various subunits of G-proteins (Oldham and Hamm 2008). GPCRs interact with the β-arrestin (ARRB) family to regulate a second wave pathway and desensitization of signaling. The β-arrestin family includes four members that are adapter proteins in the cytosol and commonly interact with various GPCRs (Lefkowitz et al. 2006). A luciferase complementation assay (LCA) based on such interactions can be used to investigate protein–protein complementation, particularly GPCRs and β-arrestin 1 (ARRB1) with emerald luciferase (Eluc) derived from click beetle (Brazilian *Pyrearinus termitilluminans*), which has fragmented N- and C-termini, and termed as a split luciferase (Hida et al. 2009; Misawa et al. 2010). As ARRB1 is a representative of ARRB family and can interacts with various GPCRs, a universal strategy can be developed for LCAs detecting interactions of various GPCRs and ARRB1 in vitro and in vivo (Misawa et al. 2010; Hattori et al. 2013; Takakura et al. 2012). In this study using a combination of the LCA and our transferable MAC, we focused on parathyroid hormone 2 receptor (PTHR2) and prostaglandin E2 receptor 4 (hEP4), which interact with (ARRB1) and are related to osteogenesis and inflammation, as an example of the numerous GPCRs. A target protein, such as a GPCR (PTHR2 or hEP4), is fused with C-terminal fragments of emerald luciferase (PTHR2-CELuc or hEP4-CEluc) and ARRB1 is fused with N-terminal fragments of emerald luciferase (NEluc-ARRB1). The fused luciferase fragments complement following the interaction of the GPCR and ARRB1, recovering their bioluminescence activity with the interaction of GPCRs and ARRB1 converted to brightness of the associating luciferase (Fig. 1a). However, this assay requires stable and simultaneous expression of these two proteins. Here, we report a dual gene expression system using a MAC and SIM system for development of an LCA to evaluate the activity of interactions of PTHR2 or hEP4 and ARRB1 by bioluminescence.

## Materials and methods

### Plasmid construction

First, the NELuc-ARRB1 expression vector, loxP_BxbiP_3HPRT_inspB4_PtNG415-ARRB1ins, was constructed by the following steps: The Vec0 fragment was prepared by digestion with SpeI and BsrGI. The fragment was ligated to inspB4ins that was digested with Acc65I and NheI. This vector was named loxP_BxbiP_3HPRT_inspB4ins. Then, loxP_BxbiP_3HPRT_inspB4ins was digested with MluI and SnaBI, and ligated to the fragment of pcDNA3.1(+)_myc-HisB_PtGRN415-ARRB1 that was prepared by digestion with MluI and PvuI. This vector was named loxP_BxbiP_3HPRT_inspB4_PtNG415-ARRB1ins. Second, the PTHR2-CEluc expression vector, pBG2-v1b1 ins PTHR2 ins, was constructed by the following steps. The pBG2-v1b1 fragment (Suzuki et al. 2014) was prepared by digestion with SpeI and MluI. The fragment was inserted into inspB4ins that was digested with NheI and AscI. This plasmid was named pBG2-v1b1_inspB4ins. Then, the two fragments of PTHR2-linker20-PtGRC394 in pcDNA4 V5_His(B) digested with BglII and EcoRI or EcoRI and PvuII were inserted into pBG2-v1b1_ inspB4ins that was digested with BamHI and SnaBI. This plasmid was named pBG2-v1b1_ ins PTHR2 ins. Third, the hEP4-CEluc expression vector, pBG2-v1b1 ins hEP4 ins, was constructed by the following steps: pBG2-v1b1_inspB4ins was digested with BamHI and SnaBI and ligated to hEP4-linker20-PtGRC394 in the pcDNA4 V5_His(B) fragment digested with BglII and PvuII (pBG2-v1b1_ ins hEP4 ins). The Bxb1-expression vector was constructed as previously described (Yamaguchi et al. 2006).

### Cell culture

CHO cells containing MAC4 (CHO/MAC4) were cultured in Ham’s F12 medium (Wako, Osaka, Japan) supplemented with 10% fetal bovine serum (FBS) (Biowest, Vieux Bourg, France), 1% penicillin/streptomycin (Wako), and 800 µg/mL hygromycin B (Wako) (Takiguchi et al. 2012; Narai et al. 2015). CHO/MAC4 cells containing a reconstructed hypoxanthine–guanine phosphoribosyl transferase (HPRT) gene and desired genes were selected in Ham’s F12 medium with 2% hypoxanthine-aminopterin-thymidine (HAT) medium (Sigma-Aldrich, St. Louis, MO, USA). HEK293 cells purchased from the American Type Culture Collection (catalog number CRL-157, Manassas, VA, USA) were cultured in Eagle’s minimum essential medium (MEM) (Sigma-Aldrich, St. Louis, MO, USA) supplemented with 10% FBS, 1% MEM non-essential amino acids (Thermo Fisher, Waltham, MA, USA), and 1% l-glutamine (Thermo Fisher). HEK293 cells containing MAC4 with the desired genes were selected in Eagle’s MEM containing 200 µg/mL hygromycin B.

### Construction of a hemagglutinin-conjugated anti-CD9 single chain antibody (ScFv) derived from the measles virus

As HEK293 cells were analyzed by flow cytometric analysis for detecting the expression of CD9 and CD46, microcell-mediated chromosome transfer (MMCT) with measles virus envelop protein (MV-MMCT) was applied and retargeted the trophism by fusion of an ScFv against CD9 (Katoh et al. 2010; Hiratsuka et al. 2015).

### MMCT via hemagglutinin and a fusion protein derived from the measles virus

A total of 1 × 10^7^ donor CHO cells were co-transfected with 12 µg of pTNH6-H-αCD9 and 12 µg of pCAG-T7-F using Lipofectamine 2000 (Thermo Fisher), according to the manufacturer’s instructions. Twenty-four hours after the transfection, the transfected cells were expanded in three T-25 flasks (Thermo Fisher) with Ham’s F12 medium for cell culture. After another twenty-four hours from the expansion, the transfected cells were cultured in Ham's F12 medium containing 20% FBS and 0.1 µg/mL colcemid (Thermo Fisher) at 37 °C for 48 h to induce micronucleation. The culture medium was changed, and the cells were incubated for another 24 h. The T-25 flasks containing CHO cells with micronuclei were filled with Dulbecco’s modified Eagle’s medium containing 10 µg/mL cytochalasin B (Sigma-Aldrich) and then centrifuged for 1 h using an Avanti HP-26XP, JLA-10,500 rotor (Beckman Coulter Life Sciences, Indianapolis, IN, USA) at 11,900×*g* to form microcells. The pellet including microcells was collected and filtered through 8-, 5-, and 3-µm pore size filters to purify the microcells. Microcell pellets were collected by centrifugation at 760×*g* in a table-top centrifuge (Kubota Corporation, Tokyo, Japan). To introduce the MAC into HEK293 cells, 2 × 10^6^ HEK293 cells were cultured in a 6-cm dish (Corning, Corning, NY, USA). The purified microcells were co-cultured with HEK293 cells. After 24 h of the co-culture, the HEK293 cells were subcultured into three 10-cm dishes. The next day, drug selection was started with 200 µg/mL hygromycin B. About 21 days later, drug-resistant colonies were picked up and expanded for the following analyses.

### Fluorescence in situ hybridization (FISH)

Metaphase chromosomes were prepared from colcemid-treated cell cultures by hypotonic treatment with 0.075 M KCl and methanol/acetate (3:1) (Wako) fixation. FISH was carried out using mouse Cot-1 DNA labeled with digoxigenin (Roche, Basel, Schweiz) and the inserted plasmid vector, loxP_BxbiP_3HPRT_inspB4_PtNG415-ARRB1ins, and pBG2-v1b1_ ins PTHR2, which were targeted to the chromosome fragment labeled with biotin (Roche). The DNA probes were labeled with a nick translation kit (Roche). Digoxigenin-labeled DNA probes were detected with an anti-digoxigenin-rhodamine complex (Roche), and the biotin-labeled DNA was detected using avidin-conjugated fluorescein isothiocyanate (Roche). The chromosomes were counterstained with 4′,6-diamidino-2-phenylindole (Sigma-Aldrich). Metaphase images were captured digitally with a CoolCubeI CCD camera mounted on a fluorescence microscope (Axio Imager, Z2; Carl Zeiss, Oberkochen, Germany). Images were processed using ISIS software provided with the microscope.

### DNA transfection for insertion of plasmid vectors using the SIM system

The ARRB1 expression vector, loxP_BxbiP_3HPRT_inspB4_PtNG415-ARRB1ins, was inserted into MAC4. Then, 2 × 10^6^ CHO/MAC4 cells were transfected with 8 µg loxP_BxbiP_3HPRT_inspB4_PtNG415-ARRB1ins and 1 µg Cre-expression vector (Invitrogen, Carlsbad, CA, USA) in a 6-cm dish using Lipofectamine 2000. After 24 h, the transfected cells were subcultured into six 10-cm dishes and incubated for a further 24 h. Then, 2% HAT medium was added to select cells with reconstitution of the HPRT gene. About 14 days later, drug-resistant clones were picked up and expanded for the following analyses.

pBG2-v1b1_ins PTHR2 was transfected into cells containing ARRB1-MAC4. Then, 2 × 10^6^ cells in a 6-cm dish were transfected with 8 µg pBG2-v1b1_ ins PTHR2 and 1 µg Bxb1 integrase expression vector using Lipofectamine 2000. After 24 h, the transfected cells were selected in medium containing 800 µg G418 (Promega, Madison, WI, USA) and 2% hypoxanthine-thymidine medium (Sigma-Aldrich) to decrease cytotoxicity of aminopterin remaining in the cells. About 15 days later, drug-resistant clones were picked up and expanded for the following analyses.

### Luciferase complementation assay (LCA)

A total of 6 × 10^4^ cells were expanded in each well of 96-well plate. The cells were stimulated with a GPCR ligand expressed in HEK293 cells. Then, the medium was removed, and the cells were cryopreserved at − 80 °C. Measurement of luciferase activity was performed with Emerald Luc Luciferase Assay Reagent Neo (Toyobo, Osaka, Japan), according to the manufacturer’s instructions. Bioluminescence was detected by an EnVision (PerkinElmer, Waltham, MA, USA). Time-lapse analysis measured the bioluminescence every 5 min. Each well was measured three times and data were corrected for average bioluminescence activity, and the data were expressed as means ± Standard Error (SE). Student’s *t*-test was used to determine statistically significant differences.

### Genomic PCR analysis

DNA was extracted with a Gentra Puregene cell kit (Qiagen, Germantown, MD, USA). PCR analysis was performed with ExTaq or LA Taq kits (TAKARA Bio Inc, Kusatsu, Japan). The following primer pairs were used for detection of each amplicon. HPRT junction: Trans-L1 (5′-TGGAGGCCATAAACAAGAAGAC-3′) and Trans-R1 (5′-CCCCTTGACCCAGAAATTCCA-3′); ARRB1: GPCR ARRB1 Fw (5′-ACGCACAGAATTCCGCTTGTGGATCTT-3′) and GPCR ARRB1 Rv (5′-GGCAACGAGTCCCTTTCCTACCAGGAG-3′); Bxb1 SIM junction: SIM HPRT Fw (5′-TGGAGGCCATAAACAAGAAG-3′) and SIM Neo Rv (5′-CGCCTTGAGCCTGGCGAACA-3′); PTHR2-CEluc: PTHR2 Fw (5′-GGAGCAGATTGTCCTTGTGCTGAAAGC-3′) and PTHR2 Rv (5′-CACGTTCCTGGGGATAGAGTCCACGAA-3′); hEP4-CEluc: hEP4 Fw (5′-CTCTGGGTTCCAGGTTCCACTGGTGAC-3′) and hEP4 Rv (5′-ATCTTGCCTGTCACGTTCCTGGGGATA-3′).

## Results

### Construction of ARRB1-MAC4 in CHO cells and evaluation of the LCA in HEK293 cells

**Fig. 2:**
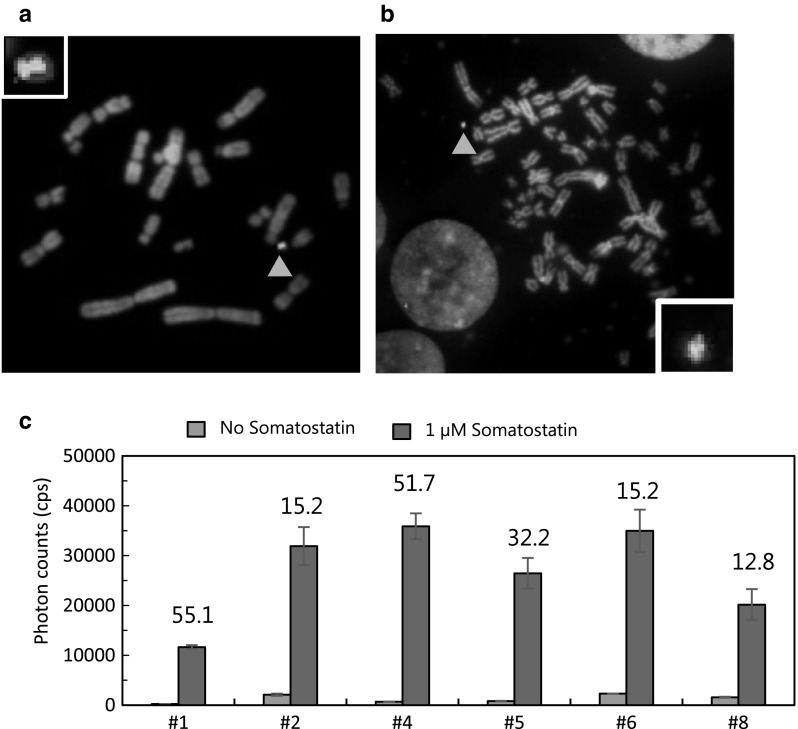
Evaluation of luciferase activity of Eluc-N-ARRB1 expressed from the MAC vector in CHO and HEK293 cells. **a** Representative metaphase of CHO cells containing MAC4 loaded with the NEluc-ARRB1 expression unit (clone #26). Red: mouse cot-1 indicates the MAC; Yellow (merged green): NEluc-ARRB1 plasmid vector probe indicates the presence of NEluc-ARRB1 DNA. Red arrowhead shows the MAC. Inset: an elongated MAC vector. **b** Representative metaphase of HEK293 cells containing MAC4 loaded with the NEluc-ARRB1 expression unit (clone #5). **c** Measurement of luciferase activity in HEK293 clones containing ARRB1-MAC4. The cells were transiently transfected with SSTR2-CEluc and treated with/without 1 µM somatostatin for 20 min. Then, the luciferase activity was measured. Blue bars: background photon counts without stimulation by the ligand. Red bars: signal photon counts with stimulation by the ligand. The number above the bar is the signal/background (S/B) ratio. Error bars: standard error of the mean (n = 3). (Color figure online)

Schematic diagrams of this study are shown in Fig. 1. At step 1, the NEluc-ARRB1 expression vector was inserted into MAC4 using the Cre-loxP system in CHO cells (Fig. 1b). Numerous HAT-resistant colonies appeared, and 26 colonies were randomly picked up for the following experiments. Three clones contained ARRB1-MAC4 independently from host chromosomes (Fig. 2a) with a high maintenance ratio (> 90%) (data not shown). CHO cells containing ARRB1-MAC4 were used for MV-MMCT towards HEK293 cells. Following the MV-MMCT, HEK293 cells were selected with hygromycin B. Nine HEK293 clones containing ARRB1-MAC4 were obtained, and PCR analysis showed that eight of the nine clones contained the NEluc-ARRB1 gene. Further FISH analysis revealed that the eight clones maintained a more than 85% retention ratio of ARRB1-MAC4 that was maintained independently from the host chromosome (Fig. 2b). To investigate NEluc-ARRB1 gene expression, SSTR2, which is a GPCR expression vector, was transfected into six clones of HEK293 cells with good growth, and then the LCA assay was performed (Fig. 1b). The results showed that all six clones had the signal of complementation of Eluc, and clone #4 showed the highest photon count of ~ 35,000 and a high signal to background (S/B) ratio of 51.7 upon stimulation with 1 µM somatostatin for 20 min (Fig. 2c). Thus, ARRB1-MAC4 was responsible for ligand signaling via GPCR.

### Construction of ARRB1-PTHR2-MAC4 in CHO cells and evaluation of the LCA in HEK293 cells

**Fig. 3:**
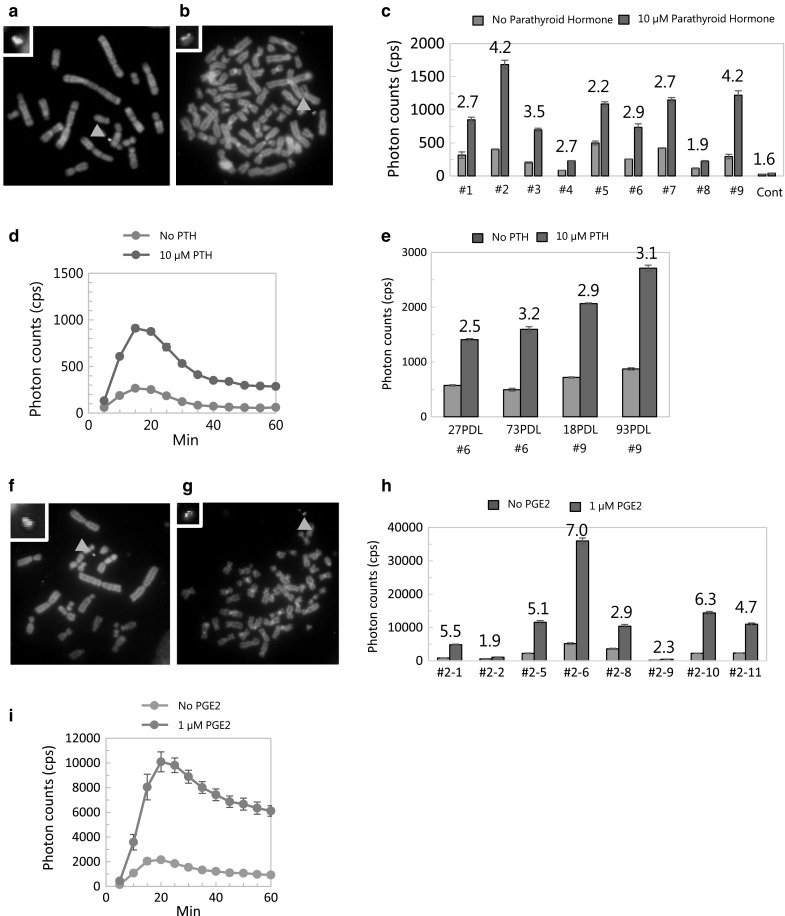
Evaluation of the LCA of NEluc-ARRB1 and PTHR2-CEluc or hEP4-CEluc expressed from the MAC in CHO and HEK293 cells. **a** Representative metaphase of CHO cells containing MAC4 loaded with NEluc-ARRB1 and PTHR2-CEluc expression unit (ARRB1-PTHR2-MAC4) (clone #9). **b** Representative metaphase of HEK293 cells containing ARRB1-PTHR2-MAC4 (clone #9). Red: mouse cot-1 indicates the MAC; Yellow (merged green): PTHR2-CEluc plasmid vector probe indicates the presence of PTHR2-CEluc DNA. Red arrowhead shows the MAC. Inset: an elongated MAC. **c** Measurement of luciferase activity in HEK293 clones containing ARRB1-PTHR2-MAC4. Cells were treated with/without 10 µM PTH for 60 min, and then luciferase activity was measured. **d** Time-lapse measurement of bioluminescence in HEK293 cells every 5 min. **e** Expression stability assay by long-term culture (PDL = passage doubling level). **f** Representative metaphase of CHO cells containing MAC4 loaded with NEluc-ARRB1 and the hEP4-CEluc expression unit (ARRB1-hEP4-MAC4) (clone #2-1). Red: mouse cot-1 indicates the MAC; Yellow (merged green): hEP4-CEluc plasmid vector probe indicates the presence of hEP4-CEluc DNA. Red arrowhead shows the MAC. Inset: an elongated MAC. **g** Representative metaphase of HEK293 cells containing ARRB1-hEP4-MAC4. **h** Measurement of luciferase activity in HEK293 clones containing ARRB1-hEP4-MAC4 (clone #2-11). Cells were treated with/without 1 µM PGE2 for 20 min, and then luciferase activity was measured. **i** Time-lapse measurement of bioluminescence every 5 min. Blue bars: background photon counts without stimulation by the ligand. Red bars: signal photon counts with stimulation by the ligand. The number above the bar is the S/B ratio. Error bars: standard error of the mean (n = 3). (Color figure online)

At step 2, CHO cells containing ARRB1-MAC4 were transfected with PTHR2-CEluc and Bxb1 integrase expression vectors to construct ARRB1-PTHR2-MAC4 and selected with G418 (Fig. 1b, c). Correctly inserted cells expressed the neomycin resistance gene, and 21 colonies were obtained. PCR analysis revealed that four of the 21 clones showed correct recombination of Bxb1 attP and Bxb1 attB as well as insertion of PTHR2-CELuc. FISH analysis showed that one clone maintained a single MAC independently from host chromosomes (Fig. 3a). The clone was used for transfer via MV-MMCT towards HEK293 cells. After drug selection with hygromycin B, 10 colonies of HEK293 cells were obtained and analyzed by PCR. The results indicated that all 10 clones contained NEluc-ARRB1 and PTHR2-ElucC. Further FISH analysis showed that nine of the 10 clones maintained the MAC independently from host chromosomes with a high retention rate of the MAC (Fig. 3b). The LCA was performed in the nine clones with 10 µM PTH for 60 min. The results showed that all clones emitted bioluminescence in response to PTH stimulation (Fig. 3c). Clone #2 showed the highest photon count of ~ 1800 and clone #9 showed the highest S/B ratio of 4.2. Time-lapse measurement of bioluminescence in clone #9 confirmed the reaction against PTH stimulation throughout the time course (Fig. 3d). Clones #6 and #9 were used to evaluate the expression stability by long-term culture. The results showed that the function of the MAC was maintained for more than 93 PDL (Fig. 3e).

In addition to construction of ARRB1-PTHR2-MAC4, hEP4-CEluc and Bxb1 integrase expression vectors were transfected into CHO cells containing ARRB1-MAC4 (Fig. 1b, c). Two clones were picked up and confirmed to have correct plasmid insertion by PCR analysis. FISH analysis showed that the two clones maintained a single ARRB1-hEP4-MAC4 independently from host chromosomes (Fig. 3f). Next, ARRB1-hEP4-MAC4 was transferred into HEK293 cells by MV-MMCT, and 12 clones were obtained after drug selection with hygromycin B. Nine clones among the 12 clones were found to maintain the correct ARRB1-hEP4-MAC4 by PCR analysis. Eight clones proliferated well and were subjected to FISH analysis. These clones maintained a single ARRB1-hEP4-MAC4 independently from host chromosomes. The LCA was performed in the eight clones with 1 µM PGE2 for 20 min. The results showed that six clones emitted bioluminescence in response to the PGE2 stimulation. Among the analyzed clones, #2-6 showed the highest photon counts of ~ 35,000 and an S/B ratio of ~ 7.0. Time-lapse measurement of bioluminescence confirmed the reaction against PGE2 stimulation throughout the time course (Fig. 3h).

In conclusion, LCAs for two GPCRs were developed using a split luciferase and SIM system for gene loading into a MAC.

## Discussion

Previously, we reported a SIM system in which three fluorescent protein genes were inserted into a HAC. Here, we report the use of the SIM system with a MAC to develop LCAs for GPCR-ARRB1 insertion and chromosome transfer of the MACs into HEK293 cells for practical application of LCAs. Because ARRB1 is generally known as a counterpart of various GPCRs, an LCA for other GPCRs can be developed by simple insertion of a targeted GPCR-CEluc into ARRB1-MAC4 using the SIM system. Therefore, we inserted an ARRB1 expression unit in the first step (Fig. 2). ARRB1-MAC4 was transferred into HEK293 cells, and the function of ARRB1 was confirmed by transfection of a plasmid expressing SSTR2, which had already been used for the LCA (Fig. 2c) (Misawa et al. 2010). Each GPCR-CEluc expression unit was inserted based on ARRB1-MAC4 in CHO cells and transferred into HEK293 cells (Fig. 3). Thus, the LCAs for PTHR2 and hEP4 were performed in HEK293 cells (Fig. 3e, h).

The constructed MAC can be transferred to various cell lines. This is the first report of chromosome transfer from CHO cells to HEK293 cells. To introduce the MAC into HEK293 cells, 2 × 10^6^ HEK293 cells were cultured in a 6-cm dish. Then, we obtained eight clones of HEK293 ARRB1-MAC4, 10 clones of HEK293 ARRB-1-PTHR2-MAC4, and nine clones of HEK293 ARRB1-hEP4-MAC4. Thus, the MMCT efficiency was ~ 2 × 10^−5^ for each chromosome transfer experiment.

Generally, random integration of an expression vector often leads to unpredictable amplification of the copy number and disruption of the expression cassette, resulting in various expression levels of unexpected patterns and even silencing. In this system, long-term gene expression was maintained with higher expression levels as a function of passages (Fig. 3e). Because the selectable marker and GPCR expression unit were loaded closely, these genes may relate to each gene expression level via the chromatin structure. Therefore, it is possible that cells with higher gene expression levels of selectable markers and the GPCR unit were enriched during long-term culture.

Each result of the LCA for ARRB1, PTHR2, or hEP4 showed different sensitivities. The HEK293 cell clones, which were transferred with each MAC (ARRB1, PTHR2, or hEP4), showed different sensitivities in the LCA (Figs. 2c, 3c, h). Because the host HEK293 cells were re-cloned via MMCT, the mRNA expression and protein synthesis ability of the host cells may be different from each clone. Therefore, clones showing higher expression levels in the LCA should be screened for practical use.

A MAC and MMCT enable introduction of an identical gene delivery vector independently from host chromosomes without the influence of chromatin modification from integrated sites on host chromosomes (Oshimura et al. 2014). Therefore, this technique allows a more accurate comparison of GPCR activities between cell lines.

This system includes a further gene integration site, ϕC31 attP, which can be recombined with ϕC31 attB by ϕC31 integrase. Importantly, the Bxb1 attB/attP and ϕC31 attB/attP reaction is irreversible unlike the Cre-loxP system. The gene-loading system using the SIM system with a HAC/MAC can be used in not only CHO cells but also HEK293 cells, although knockout of the HPRT gene in HEK293 cells is required. This system is useful to introduce multiple genes into a desired cell line by generation of the cell line containing the MAC in advance. Thus, the SIM system is convenient for gene loading of multiple genes into MAC and development of a general GPCR-ARRB1 complementation assay using a split luciferase with simultaneous loading in a single MAC.
